# Upsurge of human rhinovirus infection followed by a delayed seasonal respiratory syncytial virus infection in Thai children during the coronavirus pandemic

**DOI:** 10.1111/irv.12893

**Published:** 2021-08-04

**Authors:** Ilada Thongpan, Preeyaporn Vichaiwattana, Sompong Vongpunsawad, Yong Poovorawan

**Affiliations:** ^1^ Center of Excellence in Clinical Virology, Department of Pediatrics, Faculty of Medicine Chulalongkorn University Bangkok Thailand

**Keywords:** children, coronavirus, infection, respiratory syncytial virus, rhinovirus

## Abstract

**Background:**

Respiratory syncytial virus (RSV) and human rhinovirus (HRV) commonly cause influenza‐like illness in young children. The global coronavirus pandemic beginning in 2020 altered the seasonality and prevalence of these respiratory infections in Thailand. We aimed to characterize the upsurge of HRV and the subsequent RSV infection observed among young children who sought medical care at a hospital in Bangkok.

**Methods:**

From July to December 2020, nasopharyngeal swabs from children ≤5 years of age presented with influenza‐like illness were tested for RSV and HRV using reverse‐transcription polymerase chain reaction. Positive samples were Sanger sequenced. Genotyping was performed using sequence and phylogenetic analysis.

**Results:**

Upsurge of HRV infection began in July and was subsequently replaced by a surge of RSV infection from September onward. In 6 months, HRV was detected in 27.5% (158/574) of the samples, of which 44% (69/158) were HRV‐A, 7% (11/158) were HRV‐B, and 36% (57/158) were HRV‐C. Meanwhile, RSV was detected in 40.4% (232/574) of the samples, of which 78% (181/232) were RSV‐A and 6% (14/232) were RSV‐B. RSV peaked in October 2020, approximately 2 months later than typically seen in previous years. All RSV‐A were of subgenotype ON1. Codetection of HRV and RSV was found in 5.1% (29/574).

**Conclusions:**

HRV and RSV infection among young children coincided with relaxed local coronavirus public health measures, including the return to in‐class schooling. The delayed RSV season in 2020 was predominantly associated with RSV‐A.

## 1 BACKGROUND

Infections by respiratory syncytial virus (RSV) and human rhinovirus (HRV) commonly cause influenza‐like illness (ILI) in infants and young children.^1^ A recent study suggests that the majority of children has had at least one RSV infection by 2 years of age.^2^ Severe bronchiolitis and pneumonia from RSV infection can lead to death, which contributes to approximately 5% of global mortality in children under the age of five.^3^ Although HRV infection is generally not as severe as RSV infection, HRV is nevertheless commonly detected in the nasal and throat swabs from hospitalized young children with wheezing and from infants with pneumonia.^4^, ^5^ It also causes the common cold, and infection can often lead to acute lower respiratory tract infection and exacerbate chronic pulmonary diseases such as asthma.^6^ Treatment options for RSV or HRV are limited, and the circulation of multiple strains of these viruses has hindered the development of effective vaccines.

In Thailand, RSV infection typically peaks during July to October, which coincide with the yearly rainy season.^7^, ^8^ Annual RSV epidemics are cyclical and are associated with increased rainfall and humidity.^9^ While both genotypes of RSV (A and B) can co‐circulate within a given season, HRV prevalence is year‐round with an expected uptick early in the rainy season.^10^ Three species of HRV are A, B, and C, which have 80, 32, and 57 types, respectively.^11^

Due to the global pandemic of the coronavirus virus disease beginning in 2019 (COVID‐19), public health measures implemented to curb the transmission of COVID‐19 has reduced the incidence of pediatric hospitalization associated with certain respiratory illness in many countries including Thailand.^12^, ^13^, ^14^, ^15^, ^16^, ^17^, ^18^, ^19^ These policies included school closures, social distancing measures, and wearing of face masks in public places. However, the subsequent easing of public health restrictions including the return to classroom instructions led to an observed uptick of ILI among very young children in the second half of 2020. Despite the relative absence of influenza in the community, laboratory‐confirmed HRV and RSV were prevalent in children who sought medical care at a large hospital in western Bangkok. In this study, we described the epidemiology of the infection and characterized the prevalence of these viruses.

## 2 METHODS

### 2.1 Samples

Consecutively collected nasopharyngeal swabs from pediatric patients (≤5 years of age) with ILI who sought medical care at Bangpakok 9 International Hospital in western Bangkok were sent to our laboratory for testing (*n* = 574) during July–December 2020. Submitted samples included information regarding patient age and gender, but not extensive clinical information nor disease severity. The Institutional Review Board of the Faculty of Medicine of Chulalongkorn University approved this study (IRB number 609/59).

### 2.2 Virus detection

RSV was detected using an in‐house *TaqMan*‐based one‐step real‐time reverse‐transcription polymerase chain reaction (RT‐PCR) as previously described.^20^ Briefly, viral RNA was extracted from nasopharyngeal swabs by using the magLEAD 12gC automated extraction system (Precision System Science, Chiba, Japan). RSV matrix gene was amplified in a reaction containing 2‐μl RNA, 10 μmol of the primers and probe, and SensiFAST Probe No‐ROX One‐Step reagent (Bioline, London, UK) according to the manufacturer's instructions. Amplification parameters involved reverse‐transcription at 42°C for 20 min, followed by initial denaturation at 95°C for 3 min, and 50 cycles at 95°C for 10 s and 60°C for 20 s. For genotyping, RSV‐positive samples were subsequently subjected to conventional RT‐PCR to amplify the second hypervariable region in the G gene and Sanger sequencing as previously described.^21^

HRV was screened using conventional RT‐PCR to amplify the VP4/VP2 region.^10^ Complementary DNA was synthesized using ImProm‐II Reverse Transcription System (Promega, Madison, WI, USA) according to the manufacturer's instructions. RNA and random hexamers were heated at 70°C for 5 min and cooled on ice for 5 min. The reaction was incubated at 42°C for 2 h, followed by inactivation at 70°C for 15 min. The PCR conditions were initial denaturation at 95°C for 3 min, 40 cycles of 95°C for 1 min, 55°C for 1 min, 72°C for 1 min, and a final extension at 72°C for 7 min. Amplicons of 540 base pairs in size were visualized using 2% agarose gel electrophoresis and subjected to Sanger sequencing.

### 2.3 Sequence and phylogenetic analysis

Nucleotide sequences were deposited in the GenBank database under the accession numbers MW678173‐MW678574. HRV nucleotide sequences (~390 bp in length) were subjected to phylogenetic analysis with reference prototypes available in the GenBank database. Phylogenetic trees were reconstructed using the neighbor‐joining method with bootstrap values of 1000 replicates implemented in MEGA X.^22^ The partial G gene sequences of RSV in this study (nucleotide position 634–963 with respect to the prototypic A2 reference strain (accession number M74568) and B reference strain (accession number CH18537) were compared to RSV strains previously identified in Thailand from 2011–2019 and the global RSV sequences from other countries available in the GenBank database.^8^, ^20^, ^21^ Sequence alignment was performed using the Clustal X program. Phylogenetic tree was reconstructed by using the neighbor‐joining method with the Kimura 2‐parameter model and bootstrap values of 1000 replicates, also implemented in MEGA X software.

## 3 RESULTS

### 3.1 Epidemiological prevalence

**FIGURE 1:**
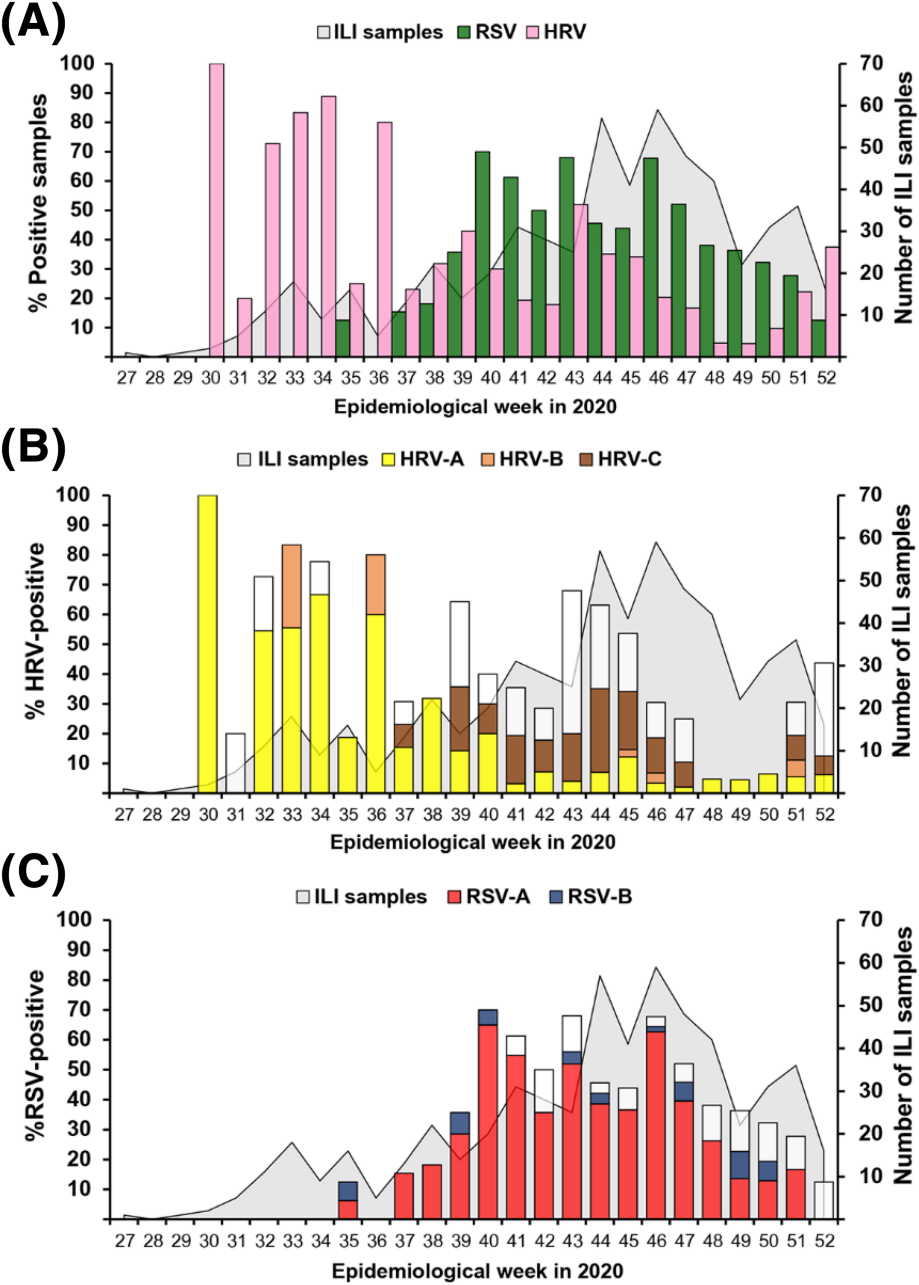
Weekly distribution of samples tested positive for RSV and HRV among medically attended children ≤5 years of age with influenza‐like illness (ILI) from July to December, 2020. (A) Overall percentage of RSV or HRV infection (left Y‐axis) relative to all ILI samples tested (right Y‐axis) during each epidemiological week (X‐axis). (B) HRV species. (C) RSV genotypes. White bars represent the untyped. Background shaded region denotes weekly ILI samples tested

From July to December 2020, a total of 574 nasopharyngeal swabs from patients with ILI were tested for HRV and RSV (Figure 1A). The age range of the patients were 14 days to 5 years (mean 2.3 years, median 2.0 years, 56% male). The majority of ILI samples arrived in the last 3 months of the year. In all, HRV was detected in 27.5% (158/574) of the samples. Of these, 44% (69/158) were HRV‐A, 7% were HRV‐B (11/158), and 36% (57/158) were HRV‐C. An additional 13% (21/158) of HRV‐positive samples could not be genotyped due to insufficient amount of PCR product. Surveillance showed that HRV began to appear in July 2020 (epidemiological week 30) and HRV‐A was initially the predominant species (Figure 1B). By September, most HRV‐positive samples were HRV‐C.

Concurrently, surveillance of RSV showed that it was infrequently detected in July and August. Beginning in September (epidemiological week 40), however, more samples tested positive for RSV than for HRV (Figure 1C). In all, 40% (232/574) tested positive for RSV by real‐time RT‐PCR, of which subsequent conventional RT‐PCR and sequencing revealed that 78% (181/232) were RSV‐A and 6% (14/232) were RSV‐B. One sample tested positive for both RSV‐A and RSV‐B. An additional 38 samples (38/232, 16%) did not yield sufficient amplicon for nucleotide sequencing. While RSV‐A infection peaked and declined during the study period, RSV‐B infection appeared sporadic. Finally, mixed infection of HRV and RSV was identified in 5% (29/574) of the samples, of which HRV‐C and RSV‐A co‐infection (7/29) comprised the majority of dual infection (Table S1). There were no differences in the mean and median ages of children who tested positive for HRV compared to those who tested positive for RSV.

**FIGURE 2:**
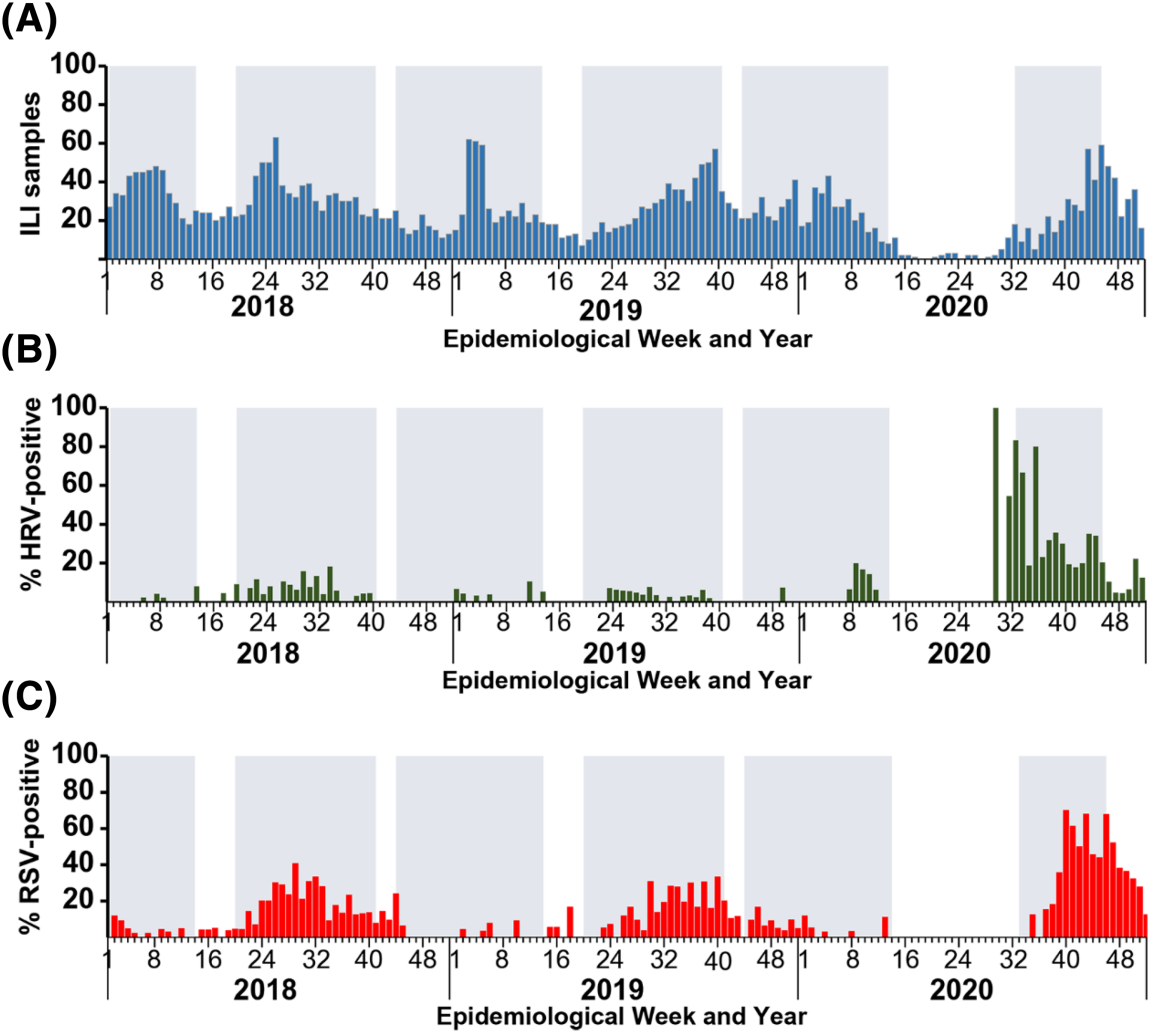
Etiology of influenza‐like illness among medically attended children ≤5 years of age during 2018–2020. (A) Total number of samples from pediatric patients with ILI testing positive for (B) HRV and (C) RSV. Block shades represent epidemiological weeks when schools were opened. Semi‐annual school closures at the end of the academic year (around epidemiological weeks 14–19) or midterm breaks (around epidemiological weeks 40–44) are unshaded

When we retrospectively examined the epidemiological trend from our data spanning 2018–2020, we observed a general increase in the number of ILI samples submitted for testing when schools re‐opened from semi‐annual breaks (Figure 2). In 2020, very few ILI samples were submitted for testing from April to June (around epidemiological week 14–26) at the height of the coronavirus pandemic in Thailand. During this time, most children were home‐bound, and school reopenings were delayed from May (typically the beginning of the academic year) to July (around epidemiological week 27). In comparison, during the first 6 months of 2020, we received 354 ILI samples for respiratory virus testing, of which fewer than 2% (6/354) and 11% (40/354) tested positive for RSV and HRV, respectively. The increase in ILI samples after epidemiological week 30 coincided with the resumption of in‐person schooling. Thus, from July to December 2020, the spike in HRV‐positive samples was followed by the surge in RSV‐positive samples, both of which occurred after schools reopened.

### 3.2 Phylogenetic analysis of HRV

**FIGURE 3:**
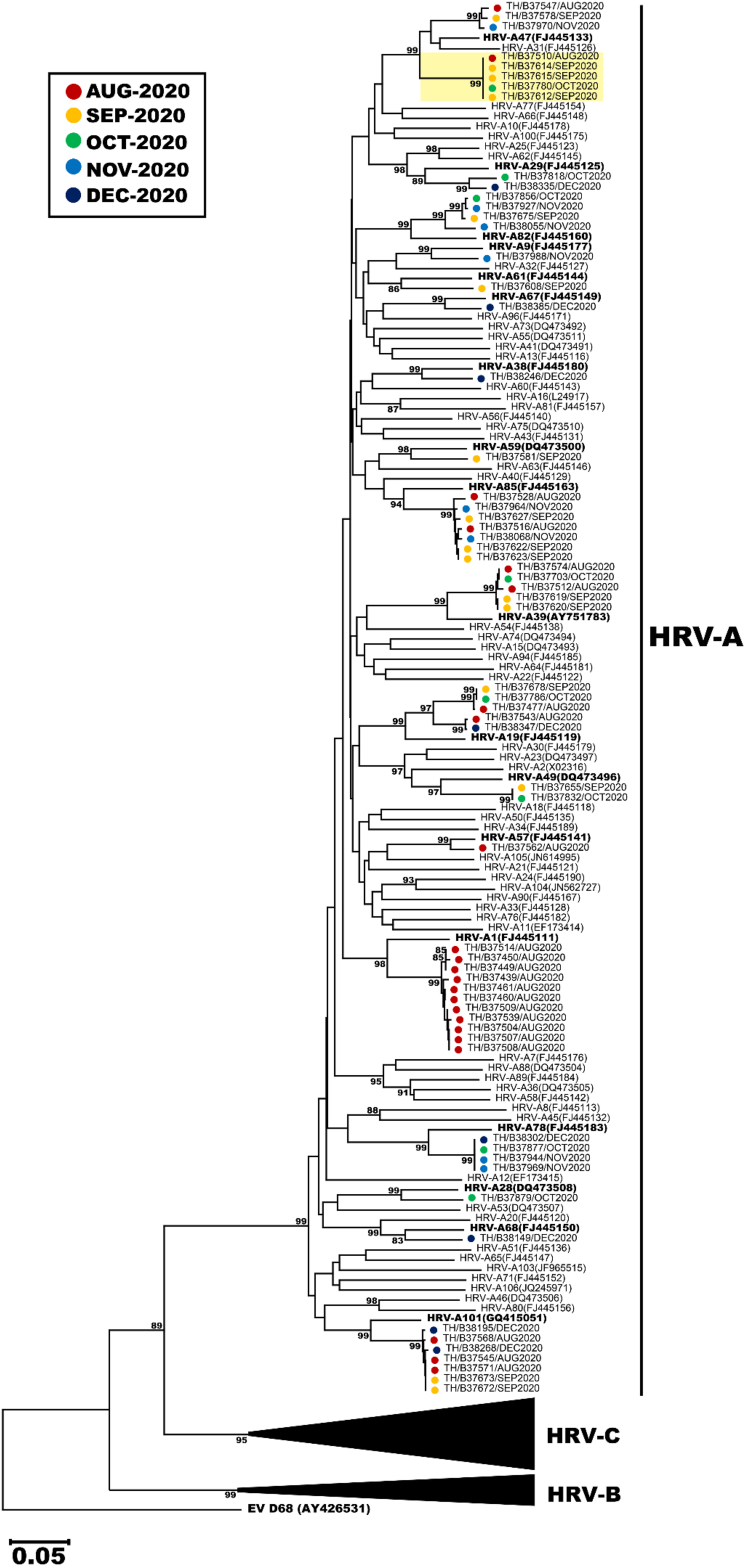
Phylogenetic analysis of the VP4/VP2 region of HRV‐A. Sequences from this study (*n* = 63) were compared to the reference HRV‐A types. The phylogenetic tree was constructed using the neighbor‐joining algorithm implemented in MEGA X. Only bootstrap values >70% are displayed at the branch nodes. Five strains which did not cluster with any reference HRV‐A are highlighted. HRV‐B and HRV‐C branches were collapsed for clarity (black triangles). Enterovirus D68 (EV‐D68) served as an outgroup. Scale bar represents nucleotide substitution rate

**FIGURE 4:**
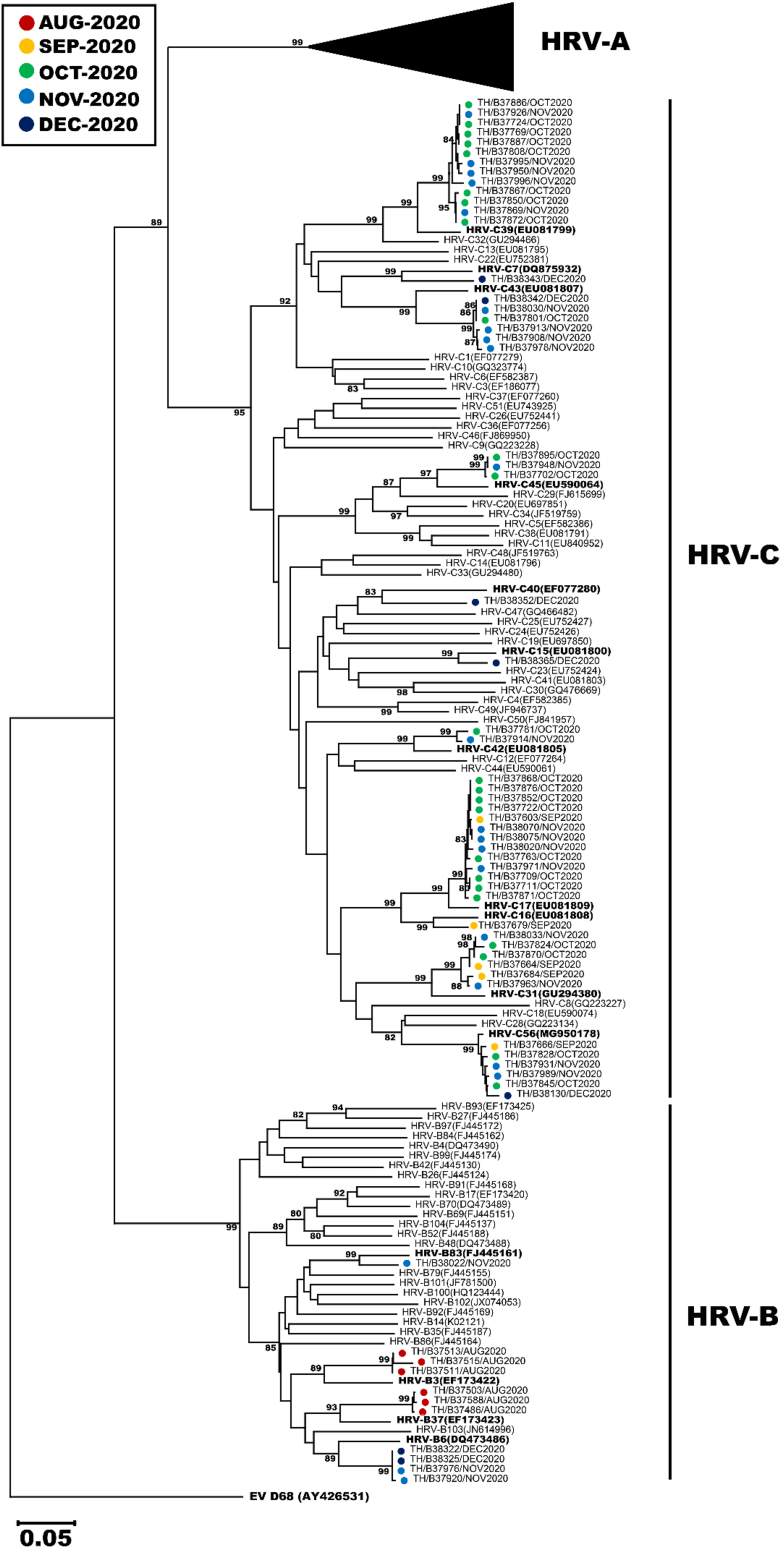
Phylogenetic analysis of the VP4/VP2 region of HRV‐B and HRV‐C. HRV‐B (*n* = 11) and HRV‐C (*n* = 53) were compared to reference types. The phylogenetic tree was constructed using the neighbor‐joining algorithm implemented in MEGA X. Only bootstrap values >70% are displayed at the branch nodes. HRV‐A branch was collapsed for clarity (black triangle). Enterovirus D68 (EV‐D68) served as an outgroup. Scale bar represents nucleotide substitution rate

To genetically characterize the HRV circulating from July–December 2020, we analyzed the VP4/VP2 sequence region of 127 strains for which we were able to obtain sufficient nucleotide sequences (63 HRV‐A, 11 HRV‐B, and 53 HRV‐C). The largest HRV‐A was type A1, which comprised 11 nonconsecutive samples identified in August 2020 (Figure 3). Other notable HRV‐A clusters were of type A85 (*n* = 7), A101 (*n* = 7), A19 (*n* = 5), A39 (*n* = 5), A78 (*n* = 4), and A82 (*n* = 4). One cluster of interest consisted of 5 strains, none of which clearly grouped with any reference HRV‐A type. Notable HRV‐B clusters were of type B6 (*n* = 4), B3 (*n* = 3), and B37 (*n* = 3) (Figure 4). Largest clusters of HRV‐C were of type C17 and C39 (*n* = 13 each), followed by C31, C43, and C56 (*n* = 6 each). When we examined the most prevalent types from each HRV species more closely, the Thai strains from this study tended to cluster together and did not show strong and distinct genetic relatedness to any particular global strains examined (Figure S1). Interestingly, we observe that HRV‐C39 tends to circulate mainly in Asian countries.

### 3.3 Phylogenetic analysis of RSV

**FIGURE 5:**
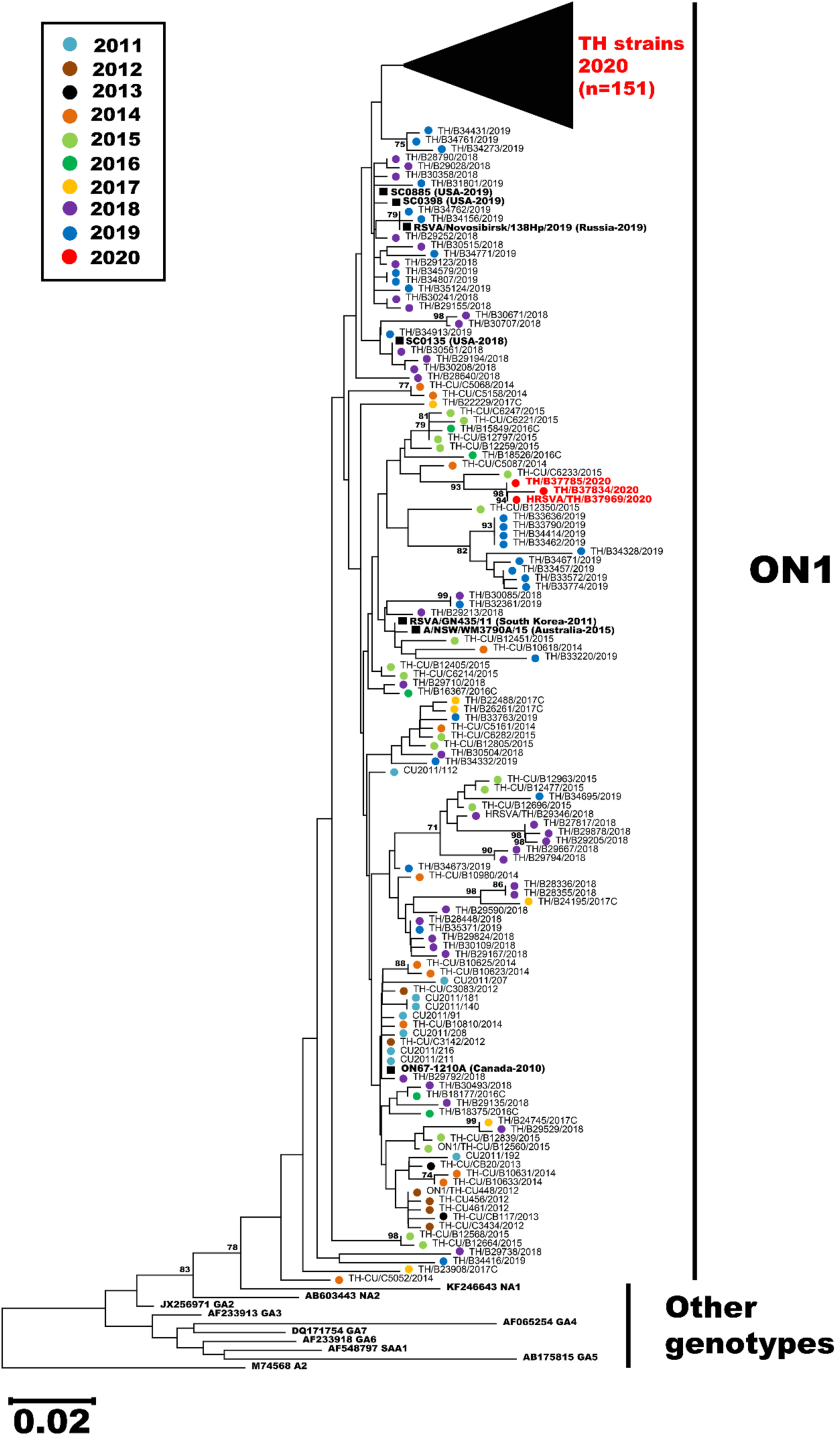
Phylogenetic analysis of RSV‐A based on the partial nucleotide sequences of the second hypervariable region of the G gene (330 bp). The phylogenetic tree was constructed using the neighbor‐joining algorithm implemented in MEGA X. Bootstrap values >70% are displayed at the branch nodes. Scale bar represents nucleotide substitution rate. Color‐coded RSV designations by year are dotted cyan (2011), brown (2012), black (2013), orange (2014), light green (2015), dark green (2016), yellow (2017), purple (2018), blue (2019) and red (2020, present study). Three 2020 strains not part of the main cluster are denoted in red. Other reference genotypes are indicated by their accession numbers

Towards characterizing the prevailing RSV strains circulating in the second half of 2020, we were able to obtain sufficient nucleotide sequences from 158 out of 232 RSV‐positive samples for phylogenetic analysis. Among these, only 4 RSV‐B sequences were adequate for phylogenetic analysis, and all belonged to the BA9 genotype. Importantly, 154 RSV‐A sequences were compared to 122 other RSV‐A strains identified in Thailand from 2011–2019 for which we have their sequence information. Phylogenetic analysis suggests that all RSV‐A strains in our study belonged to the subclade ON1 and almost all (151/154) clustered together as a separate branch, suggesting a recent divergence from previous years (Figure 5). Not surprisingly, the 2020 cluster was most closely related to the Thai strains from 2018–2019. The genetically closest global strains were the 2019 U.S. strains designated SC0398 and SC0885 (accession numbers MN306029 and MN306048, respectively), and a 2019 Russian strain designated Novosibirsk/138Hp (accession number MT422270). Three notable exceptions were the 2020 Thai strains B37785, B37834, and B37969, which grouped with older Thai strains from 2014–2016.

## 4 DISCUSSION

In the absence of specific antivirals and vaccines for RSV and HRV, monitoring the circulation of these viruses are important to better understanding their impact and infection pattern in the community. The coronavirus pandemic, which gripped many countries throughout 2020, reportedly disrupted the seasonal pattern of influenza and other respiratory illness including a decrease in seasonal influenza and a decline in hospitalization for pediatric respiratory illness.^16^, ^19^, ^23^ When the coronavirus public health measures were eased, including the resumption of in‐person schooling in the latter half of 2020, we observed an increase in childhood respiratory infection associated with HRV‐A shortly thereafter, which was eclipsed by HRV‐C a few months later. As the prevalence of HRV subsided, a surge in RSV‐associated ILI of genotype ON1 subsequently followed, albeit delayed by several months compared to previous seasons. In any given year, an upsurge of RSV infection is typically observed during the rainy season in Thailand beginning in July.^8^, ^9^ The late RSV season may be attributed to the delay in the opening of schools, which has previously been associated with the transmission of respiratory infections in the community.^24^, ^25^

Based on a previous epidemiological study conducted jointly by Thailand Ministry of Public Health and the U.S. Centers for Disease Control, RSV and HRV are the most common pathogens responsible for severe respiratory disease in young Thai children.^26^ From our surveillance of RSV over the past decade, genotype NA1 predominated prior to 2013, followed by ON1 from 2014–2015 and BA9 from 2016–2017.^8^, ^20^, ^21^ It was therefore surprising that ON1 was the only RSV‐A genotype identified in this study. Phylogenetic analysis suggests that the ON1 strains in Thailand continued to drift genetically as was evident in their clustered branch away from previously characterized strains.

Our laboratory has previously screened for and detected predominantly HRV‐A and HRV‐C in instances of pediatric lower respiratory tract infection.^10^, ^27^ Prior studies have not always defined the HRV types found in ILI samples; therefore, comparison with previous findings is often difficult. In one report, HRV‐A53 was identified in an ILI sample of unknown etiology, but overall, the group found that HRV was rarely responsible in undiagnosed respiratory infection.^28^ HRV is less seasonally dependent when compared to RSV as it tends to occur year‐round in Thailand.^19^, ^29^ The observation that HRV‐B species was least often detected was consistent with what our group and others have reported.^10^, ^29^, ^30^ It is unclear why HRV, which causes the common cold, was so frequently detected soon after schools reopened from a long recess. HRV outbreak shortly after the resumption of school has recently been reported in Hong Kong and Southampton, UK.^25^, ^30^ In those studies, it hypothesized that prolonged school dismissal may have contributed to HRV susceptibility, although this assumes that despite prior HRV exposure, waning immunity or poorly elicited immunological response could have enabled reinfection. Since HRV species and types are diverse, frequent infection in young children is certainly plausible.

Mixed viral infection of both HRV and RSV was identified in 5% of the ILI samples in this study. Our group has previously reported that infants with acute respiratory tract infection in combination with recurrent wheezing were frequently coinfected with HRV and RSV.^31^ Our finding is consistent with a previous study conducted in Thai provinces bordering Cambodia, which found these two viruses to be the most common mixed virus infection in young children hospitalized with radiographically confirmed pneumonia.^27^ Although in‐depth analysis of the mixed infection in this study would have benefited from detailed clinical information, a previous study had observed no differences in clinical symptoms and severity between HRV only, RSV only, or mixed infection in Thai patients.^29^

This study is not without limitations. Our observation relied on passive investigation of ILI samples submitted for testing. Few ILI samples in July and August could have been attributed to the low prevalence of HRV and RSV infection, or because parents were reluctant to bring sick children to the hospital for medical care during the COVID‐19 outbreak. We did not test the ILI samples for bacterial or other respiratory viruses in this study, mainly because we considered RSV and HRV to be the primary cause of respiratory infection in our cohort. Many HRV‐positive samples were unsuccessfully typed, which could have altered our conclusion that HRV‐C had displaced HRV‐A at the end of 2020. Not all children in our cohort were school‐age, and we do not know whether these children attended daycare or have school‐age siblings in the same household. Finally, observation gleamed from one sentinel hospital may not reflect the national prevalence for these viruses. Nevertheless, data from this study documents the upsurge of HRV infection among very young children, which was subsequently followed by a delayed RSV infection at the end of 2020. Characterization of HRV types and RSV genotypes circulating in the community will help to enhance epidemiological studies of two of the most important etiologies of childhood respiratory infection in developing countries.

## CONFLICT OF INTEREST

The authors declare no conflict of interest.

## AUTHOR CONTRIBUTIONS

**Ilada Thongpan:** Conceptualization; data curation; formal analysis; funding acquisition; investigation; methodology; project administration; visualization. **Preeyaporn Vichaiwattana:** Formal analysis; investigation; methodology. **Sompong Vongpunsawad:** Formal analysis; investigation; methodology; supervision. **Yong Poovorawan:** Conceptualization; funding acquisition; project administration; resources; supervision.

### PEER REVIEW

The peer review history for this article is available at https://publons.com/publon/10.1111/irv.12893.

## Supporting information


**Figure S1.** **Phylogenetic analysis of notable HRV species and types from this study.** (A) HRV‐A1 (n = 11). (B) HRV‐A85 (n = 7). (C) HRV‐A101 (n = 7). (D) HRV‐B6 (n = 4). (E) HRV‐C17 (n = 13). (F) HRV‐C39 (n = 12). Trees were constructed by using the neighbor‐joining method implemented in MEGA X. Bootstrap values >70% are indicated at the nodes. Scale bars represent nucleotide substitution rate. Colored dots denote strains identified in August (red), September (yellow), October (green), November (blue), and December (navy) of 2020.

**Table S1.** Mixed infection in 29 samples tested positive for HRV and/or RSV from July–December 2020.

## Data Availability

The data that support the findings of this study are openly available in GenBank at https://www.ncbi.nlm.nih.gov/nucleotide/, accession numbers MW678173‐MW678574.
