# Supplementary figures and images for: Upsurge of human rhinovirus infection followed by a delayed seasonal respiratory syncytial virus infection in Thai children during the coronavirus pandemic
Source: Influenza Other Respir Viruses. 2021 Aug 4;15(6):711–20. doi: 10.1111/irv.12893 (PMC8542963; doi:10.1111/irv.12893)

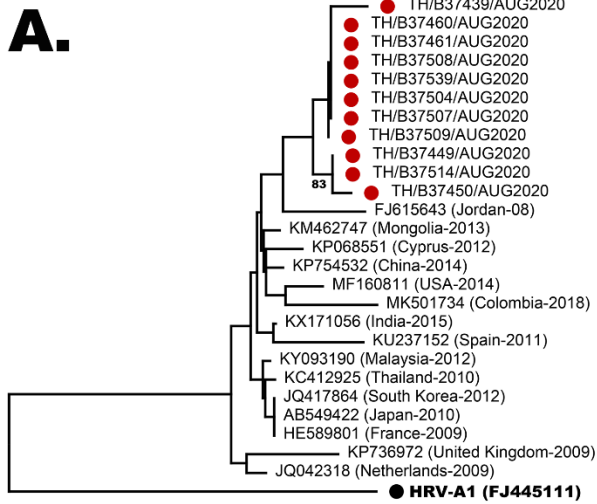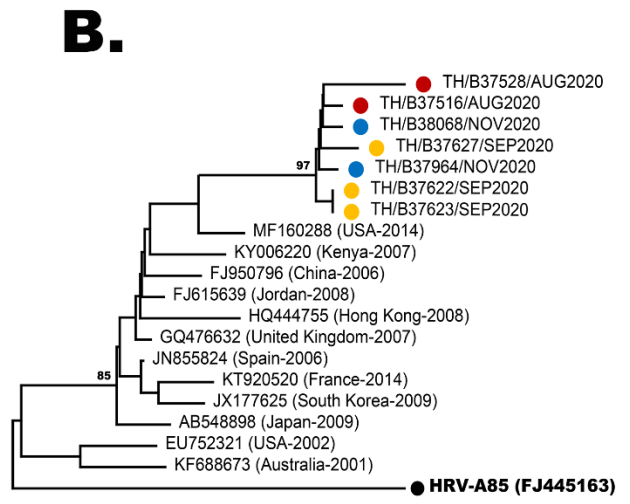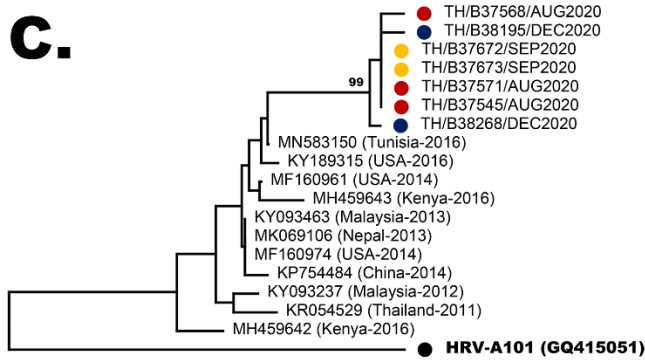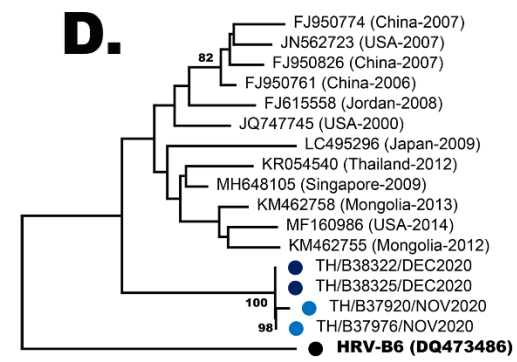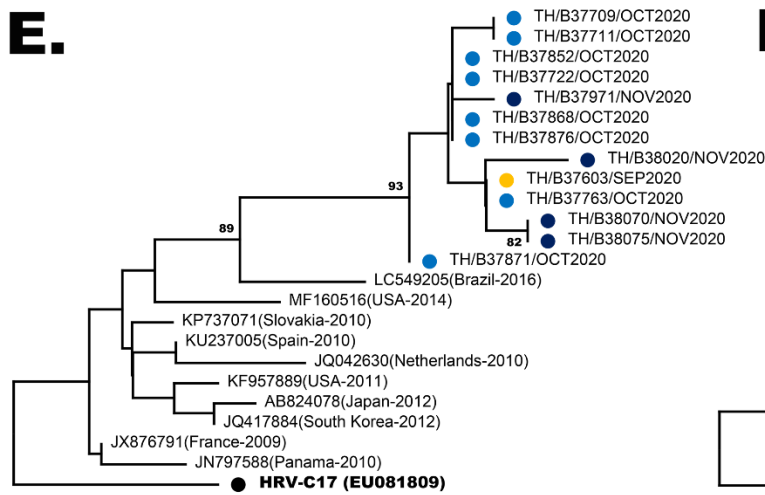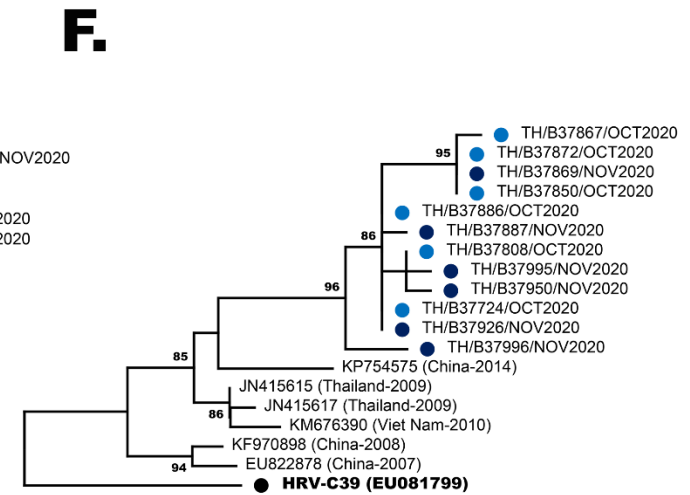

Supplement: Supplementary file 1 — Figure S1. Phylogenetic analysis of notable HRV species and types from this study. (A) HRV‐A1 (n = 11). (B) HRV‐A85 (n = 7). (C) HRV‐A101 (n = 7). (D) HRV‐B6 (n = 4). (E) HRV‐C17 (n = 13). (F) HRV‐C39 (n = 12). Trees were constructed by using the neighbor‐joining method implemented in MEGA X. Bootstrap values >70% are indicated at the nodes. Scale bars represent nucleotide substitution rate. Colored dots denote strains identified in August (red), September (yellow), October (green), November (blue), and December (navy) of 2020. [file IRV-15-711-s001.pdf]
