# Supplementary material for: Upsurge of human rhinovirus infection followed by a delayed seasonal respiratory syncytial virus infection in Thai children during the coronavirus pandemic
Source: Influenza Other Respir Viruses. 2021 Aug 4;15(6):711–20. doi: 10.1111/irv.12893 (PMC8542963; doi:10.1111/irv.12893)
Supplement: Supplementary file 2 — Table S1. Mixed infection in 29 samples tested positive for HRV and/or RSV from July–December 2020. [file IRV-15-711-s002.docx]

**Supplementary Table. Mixed infection in 29 samples tested positive for HRV and/or RSV from July-December 2020.**

|  | RSV-A | RSV (untyped) |
| --- | --- | --- |
| HRV-A | 6 (21%) | 2 (7%) |
| HRV-B | 2 (7%) | 0 |
| HRV-C | 7 (24%) | 5 (17%) |
| HRV (untyped) | 5 (17%) | 2 (7%) |
